# Development and validation of the Art Engagement Reflection Scale

**DOI:** 10.3389/fpsyg.2026.1794908

**Published:** 2026-09-22

**Authors:** Rebekah M. Rodriguez-Boerwinkle, Zorana Ivcevic, Pablo P. L. Tinio

**Affiliations:** 1 Yale Center for Emotional Intelligence, Yale University, New Haven, CT, United States; 2 Educational Foundations Department, Montclair State University, Montclair, NJ, United States

**Keywords:** aesthetic cognition, art engagement, mirror model of art, mixed-methods assessment, scale development

## Abstract

Aesthetic encounters often feel immediate, yet contemporary theories emphasize that engagement with art unfolds over time through a sequence of perceptual and cognitive processes. Despite the importance of this temporal structure to models of aesthetic experience, there are no psychometric tools designed to capture engagement as a process rather than as a disposition or an outcome. We introduce the Art Engagement Reflection Scale (AERS), a brief, mixed-methods, artwork-specific measure grounded in the mirror model of art. We present two studies that examined the psychometric properties of the AERS across controlled and naturalistic online contexts. In Study 1, participants responded to researcher-selected artworks. Confirmatory factor analyses supported a hierarchical structure in which three distinct but related levels loaded onto a higher-order depth of engagement factor. Quantitative scores showed graded engagement across levels, and qualitative analyses revealed shifts from concrete, perceptually grounded language to more abstract and interpretive content. Study 2 extended these findings to a virtual gallery context in which participants selected a personally meaningful artwork. The hierarchical structure replicated, and AERS scores showed strong convergent validity with dispositional and artwork-specific measures of aesthetic experience, as well as theoretically consistent associations with personality traits such as openness. By capturing both the degree and the nature of engagement, the AERS offers a flexible tool for testing process-oriented theories of aesthetic experience in laboratory, museum, and field settings.

## Introduction

Aesthetic encounters often feel immediate: you look at a work of art, and in return, you experience a feeling or make a judgment about it. Yet decades of theory suggest that such experiences are anything but instantaneous. Engagement with art unfolds over time and includes multiple levels of processing, starting with perceptual exploration, progressing to associations with our personal memories and learned knowledge, and concluding in more elaborated meaning-making, aesthetic judgments, and emotions.

Although this temporal structure is central to contemporary models of aesthetic experience (e.g., Chatterjee, 2004; Leder et al., 2004; Leder and Nadal, 2014; Pelowski et al., 2017; Tinio, 2013), it has not commonly been captured empirically. Existing measures of experience of art tend to focus either on stable dispositions toward art, like aesthetic sensitivity (Schlotz et al., 2021), or on the outcomes of engagement, such as absorption (Silvia and Nusbaum, 2011) or pleasure (Wanzer et al., 2020), leaving the process itself largely unmeasured.

In the present work, we address this gap by using the mirror model of art (Tinio, 2013) as a theoretical base for the development and validation of the Art Engagement Reflection Scale (AERS), a brief, mixed-methods, artwork-specific measure designed to capture the intensity and content of viewers’ engagement with art across successive levels of aesthetic processing.

### Theoretical basis for scale development: the mirror model of art

The mirror model of art (Tinio, 2013) provides a process-oriented framework for aesthetic experience that explicitly links artistic creation and aesthetic reception. The central idea of the model is that the stages involved in producing an artwork are “mirrored,” in reverse order, during its reception by viewers. Rather than treating aesthetic experience as a single evaluative moment, the model conceptualizes engagement as unfolding over time through a sequence of three qualitatively distinct, but interconnected, phases. On the side of art viewing, this loose temporal structure allows the mirror model to accommodate the overall forward progression of aesthetic engagement from early, automatic processing to deeper meaning-making and final judgment. At the same time, it also acknowledges that individuals may revisit or loop through a level multiple times as their aesthetic experience deepens. Our subsequent discussion of the mirror model in the present work focuses on this process on the side of the viewer or perceiver of artistic stimuli.

The first level of the mirror model on the side of artistic reception, *early, automatic processing*, focuses on perceptual analysis of a work of art. At this stage, viewers attend to the low-level visual characteristics of an artwork, such as the colors, shapes, and textural features present. This part of the process is largely stimulus-driven, relying heavily on visual exploration and low-level perceptual mechanisms that extract structure from the image. Although this level is often rapid and automatic, it is important for establishing the foundation for further engagement by determining what information is available for viewers to draw on.

The second level, *intermediate, memory-based processing*, involves linking perceptual features of the artwork to prior personal experiences, cultural schemas, and learned knowledge. This level is characterized by the recognition and characterization of objects, events, and other elements of the work by the viewer. While perceptual input may remain active, this process is more deliberate than Level 1 and can often involve several iterations that help expand and adapt the viewer’s understanding of the artwork. Because this level is driven by internal forces of memory in addition to external cues from the artwork, the results are more subjective and variable, depending on individual differences in art interest and expertise, as well as personal life history.

The final level of the mirror model, *meaning-making and final judgment*, concerns aesthetic and affective evaluation of the artwork. At this point, viewers consolidate their interpretations of the artwork and articulate judgment about its overall themes, messages, or value. They may also highlight insights that extend beyond the artwork itself, situating the artwork within broader emotional, cultural, or philosophical frameworks. They may also arrive at final judgments about the clarity or outstanding ambiguity of the work.

Prior research has used the mirror model primarily as a descriptive and organizing framework for understanding aesthetic experience. Specker et al. (2017) showed that viewers’ spontaneous, open-ended responses to artworks could be coded using the model’s stages, lending ecological validity to the framework and revealing that engagement is not strictly linear, with frequent movement between Levels 2 and 3. In a second study, they demonstrated that information structured to align with mirror model stages improved memory for artworks in a museum, but not in a laboratory setting. However, their reliance on qualitative methods limited the ability to quantify the degree of engagement at each stage, systematically compare engagement across individuals or artworks, or address psychometric properties such as reliability and dimensional structure. The relative scarcity of Level 1 content (likely reflecting the rapid and implicit nature of perceptual processing) also suggests that early processing may be underestimated by open-ended only approaches.

In addition, Tinio and Gartus (2018) showed that emotional characteristics of artworks correspond systematically with viewers’ emotional responses toward these artworks across multiple affective dimensions. More recently, meta-analytic neuroimaging evidence has provided support for the model’s mirrored artist-viewer structure. Vartanian et al. (2025) identified distinct but overlapping neural networks associated with the early stages of figural creativity and aesthetic appreciation of visual art. In another study of the link between creation and aesthetic appreciation, Sacheli et al. (2022) demonstrated substantial overlap between neural systems involved in aesthetic perception and visual creativity, with the strongest convergence emerging in higher-order associative and integrative networks.

Each of these studies, however, highlights a measurement gap between theory and method. Although existing research provides evidence for multiple levels of art processing and correspondence between the processes of creation and appreciation, there is a need for ways to better quantify where someone is in the art engagement process and how deeply they are engaged, as well as for ways to assess the content of art engagement as a process.

### Measuring art experience

A number of self-report measures have been developed to assess how people experience art, but these scales differ markedly in what they measure and how they are intended to be used (for an overview, see Table 1). Some instruments focus on stable dispositions, such as the *Desire for Aesthetics Scale* (DFAS; Lundy et al., 2010), which captures a trait-like motivation to seek out aesthetic experiences, or the *Aesthetic Responsiveness Assessment* (AReA; Schlotz et al., 2021), which assesses general sensitivity to aesthetic stimuli. Others emphasize state-based responses, including the *Aesthetic Experience Questionnaire* (AEQ; Wanzer et al., 2020) and the *Unusual Aesthetic Experiences Scale* (UAES; Silvia and Nusbaum, 2011), which capture outcomes of engagement such as absorption, pleasure, or rare experiential qualities elicited by a specific encounter (such as chills). Existing scales also vary in whether they are general (describing aesthetic tendencies across contexts) or artwork-specific, and in their practicality for field use—with some requiring lengthy batteries or specialized prompts that limit use in museums and other naturalistic settings.

**Table 1:** Overview of self-report measures of aesthetic experience and engagement.

Scale	Scale features			
	Trait vs. State	General vs. Art-Specific	Practicality for field use	Dispositions vs. Process vs. Outcomes
Aesthetic Experience Questionnaire (AEQ; Wanzer et al., 2020)	Primarily trait-like (retrospective)	General	Medium	Outcomes
Aesthetic Responsiveness Assessment (AReA; Schlotz et al., 2021)	Trait	General	High	Dispositions
Art Impact Taxonomy (Christensen et al., 2023)	State (descriptive)	Artwork-specific	Low	Outcomes
Desire for Aesthetics Scale (Lundy et al., 2010)	Trait	General	Medium	Dispositions
Unusual Aesthetic Experiences Scale (UAES; Silvia and Nusbaum, 2011)	Either	Either	High	Outcomes
Visitor Engagement Scale (Taheri et al., 2014)	State	Context-specific (Exhibit-level measure)	High	Outcomes
Art Engagement Reflection Scale (AERS)	State	Artwork specific	High	Process

Conceptually, most existing scales measure either dispositional inputs (what people bring to art encounters) or outcomes (what people report having experienced), with few tools aimed at characterizing the process of engagement itself. Measures such as the *Visitor Engagement Scale* (Taheri et al., 2014) and the *Art Impact Taxonomy* (Christensen et al., 2023) may capture behavioral or experiential facets of engagement *in situ*, but they are often broad, descriptive, and outcome-focused rather than theory-testing.^1^ This distinction matters because aesthetic experience is not a single feeling or judgement; it is an organized sequence of cognitive–affective operations that shape how meaning is constructed.

### Developing the Art Engagement Reflection Scale (AERS)

Amid this landscape of existing measures, the AERS was developed to capture a theory-driven process of aesthetic engagement, across multiple levels of complexity (for full scale, see Appendix 1). The AERS is designed to assess to what extent and *how* people engage with an artwork as that experience unfolds. It is an artwork-specific, state-based measure that is structured around stages of engagement derived from the mirror model of art.

Item development for the AERS was theory-driven and based directly on the cognitive processes described by the mirror model (Tinio, 2013). The first author generated an initial item pool based on the defining characteristics of each processing stage: (1) Early, automatic processing, which focuses on rapid perceptual attention to the visual features of an artwork; (2) Intermediate, memory-based processing, which reflects the integration of perceptual input with prior knowledge, personal experiences, and learned concepts; and (3) Meaning-making and final judgment, which captures broader interpretation, evaluation, and reflection on the artwork. Items were written as first-person, artwork-specific statements to ensure that the scale assessed state engagement with a particular artwork rather than general attitudes toward art. The initial item pool was iteratively refined for clarity, brevity, and theoretical specificity by the three project leads, with the goal of minimizing conceptual overlap while maintaining coverage of each processing stage. In addition to the rating scale items, one open-ended prompt was developed for each stage to capture the artwork-specific content of participants’ engagement and complement the quantitative ratings. The goal of these open-ended items was to add a qualitative assessment so that we could gauge not only the degree or intensity of engagement across the three levels, but also the nature of the engagement. For example, we could learn not only that participants engage in memory-based processing, but also to which semantic and/or episodic memories they relate the artworks (e.g., being reminded of a specific life-experience when viewing an artwork).

The AERS was developed with both basic research and practical field use in mind: it is brief (12 Likert items and 3 open-ended questions), modular, and compatible with museum and gallery contexts where participant time and attention are limited. By integrating Likert-style items with open-ended responses, the scale occupies a middle ground between quantitative only self-report measures and rich, purely qualitative approaches, providing both comparability across participants and access to the content of art-related cognition. In this way, the AERS complements existing instruments while addressing a gap in the measurement of structured, process-level engagement with art.

The development of the AERS was, in part, motivated by the need for a measure that was explicitly grounded in aesthetic processing theories in the field of empirical aesthetics (e.g., Leder et al., 2004; Tinio, 2013). A theory-driven process measure of art engagement can enable further hypothesis testing. Although previous studies have shown that viewers’ responses can be mapped onto the model’s stages (e.g., Specker et al., 2017), this evidence has relied largely on qualitative coding or indirect manipulations, limiting the ability to quantify engagement, compare individuals or artworks, or test the proposed structure of art processing psychometrically. By operationalizing processing at each stage of the mirror model as a latent factor, the AERS enables direct, level-by-level measurement of engagement, allowing researchers to test whether the stages are empirically distinct, how strongly they cohere into a higher-order construct, and how engagement looks across artworks and contexts.

Additionally, the AERS was designed as a mixed-methods instrument, combining structured quantitative items with open-ended responses. This approach allows the scale to capture both the degree of engagement at each level, and the quality and content of that engagement: both how much and what (in artwork-specific terms) viewers attend to, associate in their memory, and ultimately take away from the work. Item sets for individual levels can be used alone (e.g., only assessing deeper levels of processing), or the scale administrator can choose to only use the quantitative or qualitative portions. The modular structure of the AERS makes it adaptable for diverse research contexts, including museum studies and field-based work, where brevity and flexibility are critical.

## Present research

The development of the AERS addresses a familiar problem in empirical aesthetics. We have strong theoretical accounts which describe the structure of aesthetic experience, and we have well-established measures of aesthetic dispositions and experiential outcomes. What remains surprisingly scarce, however, are psychometric tools that directly measure the process of engagement—that is, how viewers move through different levels of processing during an encounter with a specific artwork. As a result, predictions of process-oriented models of cognition are difficult to test quantitatively, especially in naturalistic settings. The present work takes up this challenge by developing and validating a theory-driven measure designed to assess aesthetic engagement across successive stages of processing. The following sections describe two studies that aim to validate the structural and convergent properties of the AERS in one researcher-constrained and one participant-driven research scenario. Across these studies, we evaluated the psychometric structure, reliability, and interpretive validity of the AERS using both quantitative and qualitative data, demonstrating how the scale operates and how its results can be used and interpreted.

Study 1 tested the basic structure of the AERS using researcher-selected landscape artworks, examining whether responses organized into three empirically distinguishable, but related levels corresponding to the mirror model. We also assessed internal consistency and convergent validity with established measures of art-related dispositions and aesthetic outcomes. At the qualitative level, we examined whether open-ended responses exhibited systematic shifts in semantic properties across levels, consistent with theoretical predictions about movement from concrete, perceptually-grounded descriptions toward more abstract and interpretive language. We expected: (a) a graded pattern of engagement across levels, (b) that each level would contribute uniquely to a higher-order degree of engagement, and (c) qualitative evidence that the content of engagement changes in theoretically-consistent ways as viewers move through the process.

Study 2 extended this work into a more naturalistic, self-directed context by embedding the AERS in a virtual gallery environment in which participants selected an artwork that was personally meaningful to them. This design allowed us to test whether the scale functions similarly when engagement is self-initiated rather than experimentally constrained. In addition to replicating the factor structure and reliability observed in Study 1, we examined whether individual differences relevant to aesthetic engagement (e.g., openness to experience) related systematically to level-specific engagement.

Data collection for both Study 1 and Study 2 was approved under the Yale University IRB (protocol #2000039007). All participants provided informed consent.

## Study 1

### Methods

#### Participants

A total of 200 adult, English-speaking participants were recruited using the Prolific.co study recruitment platform, which is an online tool that enables researchers to recruit human participants to complete surveys and behavioral studies in exchange for participation rewards. After screening for low-quality responses (e.g., incomplete responses, duplicates, responses that were returned or denied in Prolific), a final sample of 192 participants was retained for data analysis. Participants were on average 36.8 years old, with considerable age variability (*SD* = 13.5). In terms of gender, the sample was relatively balanced: 98 identified as women, 91 identified as men, and three preferred to self-describe.

#### Materials

##### Artworks

Two artworks were selected by the first author for use in Study 1. Both artworks were obtained from the Vienna Art Picture System (VAPS; Fekete et al., 2023; for basic artwork information, see Supplementary Table 1), a dataset of 999 art images collected for empirical research in the arts. The selected images were both landscapes to control for subject matter and ensure that differences in viewer engagement could be attributed primarily to style rather than content. Within this category, the artworks were chosen to vary in terms of their abstraction: one image was highly representational, depicting a realistic village scene with clearly identifiable natural elements, while the other was a more abstract, surrealist landscape that emphasized color, form, and compositional structure over any recognizable content. This contrast was chosen to allow us to examine how aesthetic engagement unfolds when participants encounter works that differ in representational clarity, while keeping general scene content similar. Further, selecting both artworks from a standardized, research-oriented dataset also ensured consistent image quality, size, and resolution for presentation within the present study.

##### AERS

The AERS is a process measure of engagement with a work of art. 12 first-person items (four per level) measure three subprocesses: (1) Early, automatic processing (Level 1; “I noticed the fine details of the artwork quickly.”), (2) Intermediate, memory-based processing (Level 2; “As I looked at the artwork, I found myself making connections with other things I’ve seen or learned.”), and (3) Meaning-making and final judgment (Level 3; “The artwork made me reflect on deeper concepts or questions.”). Responses were provided on a 5-Point Likert scale from *Strongly Disagree* to *Strongly Agree*. After each section of quantitative items, one open-ended question about the artwork-specific content related to that level was administered, for example: “Describe the specific visual elements (e.g., colors, textures, shapes) that first caught your attention. What was it about them that stood out to you?” (Level 1).

##### Aesthetic Experiences Questionnaire (AEQ)

Wanzer et al. (2020). The AEQ contains 22 items that ask “In general, when I view art…”, followed by first-person statements (e.g., “I experience a wide range of emotions.”) that measure dispositional or characteristic experiences when encountering art. The scale can be scored using a composite, overall score representing the strength of a person’s disposition toward aesthetic experiences, or individual dimensions, which comprise traditional dimensions of aesthetic engagement (i.e., perceptual, emotional, cultural, understanding), as well as a flow component (i.e., flow-proximal conditions, flow-experience). Participants provided ratings using a 5-point Likert scale, from *Strongly disagree* to *Strongly agree*, and we summed scores across all items.

##### Unusual Aesthetic Experiences Scale (UAES)

Silvia and Nusbaum (2011). The UAES contains 12 items that measure strength of experiences like “feel[ing] absorbed and immersed”, “get[ting] goose bumps”, and “feel[ing] like crying”. The scale can be scored for three latent factors—aesthetic chills, feeling touched, and absorption—or a shared global higher-order factor of aesthetic experience. The scale is designed to be modified by the researcher so that it can be implemented in a variety of artistic domains (e.g., in response to visual art, music, film, etc.) or in response to different art-related experiences (e.g., viewing a single artwork or visiting an entire exhibit); in this spirit, we made an artwork-specific modification of the UAES. Responses were provided on a 7-point Likert scale, from *Not at all* to *Very much*.

#### Procedure

After being recruited for study participation via Prolific, participants were redirected to Qualtrics to complete informed consent and respond to study measures. First, people provided basic demographic information (age, gender) and responded to the AEQ (person-level measure). Then, in a repeated measures design, people completed the artwork-specific UAES and AERS in response to the two researcher-selected artworks whose presentation order was counterbalanced. After survey completion, participants were redirected to Prolific for a payment of $3.00 USD.

## Results and discussion

Descriptive statistics for the full sample in each study are presented in Table 2, and full-sample correlations are shown in Table 3. All participants viewed both the abstract and the representational artworks, so psychometric findings for each are provided separately.

**Table 2:** Descriptive statistics for Study 1 and Study 2.

	Study 1 (*n* = 192)	Study 2 (*n* = 298)
Age	36.8 (*13.5*)	40.7 (*12.1*)
Gender (Women, Men, Other)	98/91/3	136/159/3
Educational Attainment		“Bachelor’s degree” (*Mode* = 109)
Art Interest		2.9 (*0.75*)
Recent Gallery Visit? (% Yes)		67%
Aesthetic experiences (AEQ)	76.66 (*17.48*)	65.54 (*11.27*)
Unusual Aesthetic Experiences (UAES)	R: 32.27 (*16.07*) A: 32.06 (*16.89*)	32.33 (*9.41*)
Big Five traits		
Openness to experience		4.53 (*1.05*)
Conscientiousness		4.82 (*1.03*)
Extraversion		3.05 (*1.39*)
Agreeableness		4.92 (*1.02*)
Disagreeableness		2.50 (*1.35*)
Neuroticism		2.75 (*1.36*)
Art engagement (AERS)		
Depth of engagement (total score)	R: 37.15 (*12.56*) A: 33.34 (*12.04*)	45.61 (*8.03*)
Level 1	R: 13.27 (*4.24*) A: 13.83 (*4.05*)	16.64 (*2.68*)
Level 2	R: 12.43 (*4.84*) A: 9.31 (*4.82*)	14.22 (*3.86*)
Level 3	R: 11.36 (*4.88*) A: 10.21 (*4.77*)	14.74 (*3.60*)

Data reflect all variables administered in Studies 1 and 2. Descriptive statistics for Study 2 Aesthetic Experience Questionnaire (AEQ) were derived from a reduced sample (n = 287) due to a researcher error in survey administration. In Study 1, AERS was administered separately for the representational (R) and abstract (A) artworks; in Study 2, AERS was administered once for a personally meaningful artwork selected by participants. Values indicate M (SD) unless otherwise specified.

**Table 3:** Study 1 correlations.

	1	2	3	4	5	6	7	8	9	10	11	12
1. Age	-											
2. Gender	0.08	-										
3. Aesthetic Experiences (AEQ)	−0.15^*^	0.03	-									
Unusual Aesthetic Experiences (UAES)												
4. UAES (R)	−0.22^***^	0.00	0.53^***^	-								
5. UAES (A)	−0.30^***^	0.01	0.56^***^	0.65^***^	-							
Art Engagement (AERS)												
6. AERS Global (R)	−0.08	0.04	0.51^***^	0.75^***^	0.42^***^	-						
7. AERS Global (A)	−0.18^**^	−0.02	0.48^***^	0.46^***^	0.78^***^	0.43^***^	-					
8. AERS Level 1 (R)	0.02	0.04	0.44^***^	0.59^***^	0.27^***^	0.84^***^	0.29^***^	-				
9. AERS Level 1 (A)	−0.19^**^	0.01	0.43^***^	0.24^***^	0.59^***^	0.27^***^	0.79^***^	0.19^**^	-			
10. AERS Level 2 (R)	−0.08	0.00	0.45^***^	0.68^***^	0.40^***^	0.92^***^	0.43^***^	0.62^***^	0.27^***^	-		
11. AERS Level 2 (A)	−0.09	−0.03	0.39^***^	0.46^***^	0.67^***^	0.40^***^	0.91^***^	0.26^***^	0.55^***^	0.40^***^	-	
12. AERS Level 3 (R)	−0.14^*^	0.08	0.49^***^	0.74^***^	0.45^***^	0.94^***^	0.44^***^	0.68^***^	0.26^***^	0.85^***^	0.40^***^	-
13. AERS Level 3 (A)	−0.21^**^	−0.03	0.47^***^	0.49^***^	0.78^***^	0.46^***^	0.93^***^	0.32^***^	0.59^***^	0.48^***^	0.83^***^	0.48^***^

Individual difference and demographic variables were assessed once per participant. Artwork-specific measures were assessed separately for the representational (R) and abstract (A) artworks; correlations therefore reflect associations between stable person-level variables and engagement with each artwork considered independently. Gender was coded 0 = female; 1 = male. *p < 0.05; **p < 0.01; ***p < 0.001.

Since the quantitative portion of the AERS was designed to capture the degree of overall aesthetic engagement with a work of art, as well as the degree of engagement for each of the three viewing stages described by the mirror model (Level 1: Early, automatic processing; Level 2: intermediate, memory-based processing; Level 3: meaning-making and final judgments), scores were calculated both for the three levels and as a composite, overall score.

A hierarchical confirmatory factor analysis (CFA) was conducted to evaluate the structure of the AERS. The model included three lower-order factors corresponding to the mirror model stages as well as a higher order factor representing depth of engagement. To capture the theoretical continuity and overlap between adjacent stages of art viewing in the mirror model, the structural model allowed correlations between Level 1 and Level 2, as well as Level 2 and Level 3, but not between Level 1 and Level 3. Each lower-order factor was indicated by four items specific to that stage, and the three lower-order factors were specified to load onto a single higher-order factor representing general depth of engagement.

### Psychometric findings for the representational artwork

The composite or global score, representing overall depth of engagement across the three levels, had a mean of 37.15 (*SD* = 12.56) on a possible range of 12–60 and demonstrated high internal consistency (*α* = 0.95). At the level-specific scale (possible range of 4–20 for each level), Level 1, which assesses perceptual engagement, had a mean of 13.27 (*SD* = 4.24) with high reliability (*α* = 0.88). Level 2, assessing associative and memory-based reflection, had a mean of 12.43 (*SD* = 4.84) and demonstrated high reliability (*α* = 0.90). Level 3, which captured meaning-making and final judgments, had a mean of 11.36 (*SD* = 4.88) and also showed high reliability (*α* = 0.93). The descriptive patterns indicate that viewers engaged the researcher-chosen representational artwork in a graded fashion: engagement was strongest at the perceptual level and declined modestly as processing shifted into more interpretive and reflective territory. Each scale nonetheless demonstrated strong reliability for its intended facet of aesthetic engagement.

The hierarchical CFA demonstrated strong fit to the data for the representational artwork. Fit indices exceeded conventional thresholds, with a Comparative Fit Index (CFI) of 0.94 and Tucker-Lewis Index (TLI) of 0.93, indicating that the proposed hierarchical structure captured a substantial proportion of the observed covariance. The Standardized Root Mean Square Residual (SRMR) was excellent (0.04), suggesting that model-implied correlations closely matched the empirical correlation matrix. The Root Mean Square Error of Approximation (RMSEA; 0.11; 90% CI: [0.09, 0.13]) was somewhat higher than ideal, reflecting modest absolute misfit; however, because the sample size (*n* = 192) was relatively small for a hierarchical CFA, we also evaluated the robust RMSEA, which adjusts for non-normality and small-sample bias. The robust RMSEA (0.10; 90% CI: [0.07, 0.12]) indicated improved absolute fit and supported the adequacy of the hierarchical structure. These patterns suggest that the model provides a coherent and acceptable representation of engagement for the representational artwork.

Standardized factor loadings for all items were also strong and statistically significant, which supports the reliability of the three lower-order factors. Level 1 loadings ranged from 0.79 to 0.82, demonstrating a cohesive perceptual processing factor. Level 2 items loaded between 0.77 and 0.89, with the highest loading for the item connecting art to lived experience (i.e., “I found myself reflecting on how the artwork relates to my personal experiences”). Level 3 item loadings were the highest, ranging from 0.85 to 0.90. At the higher level, all three lower-order factors loaded robustly onto the Depth factor (standardized: L1 = 0.94, L2 = 0.85, L3 = 0.81). This pattern suggests that while Levels 1–3 each represent unique aspects of engagement, they all contribute substantially to a common underlying construct of overall depth. The correlations among the lower-level factors—non-significant and negative between Levels 1 and 2 (*r* = −0.10; *p* = 0.16), and positive and significant between Levels 2 and 3 (*r* = 0.33; *p* = 0.04)—are consistent with theorized variability in how viewers transition between early perceptual and later associative and interpretive modes when encountering representational artworks. These results reflect the theoretical prediction that not everyone moves beyond the first level of processing. But once people move to the intermediate level of processing, they are more likely to proceed to the deepest level too.

The model fit indices, standardized loadings, and adjacent factor correlations altogether demonstrate that the hierarchical model provided strong support for the conceptual structure of the AERS when it is applied to a representational artwork. The high loadings of all three levels onto the higher-order Depth factor indicate that the AERS captures a meaningful general construct of engagement that spans all levels of aesthetic processing. At the same time, the distinct and well-defined lower-order factors demonstrate that each engagement level retains unique variance, which suggests that people’s responses can meaningfully differentiate between the three stages of the mirror model.

### Psychometric findings for the abstract artwork

The overall depth of engagement composite averaged 33.34 (*SD* = 12.04) and showed high internal consistency (*α* = 0.94). At the level of the three subscales, participants reported the highest scores for Level 1 (*M* = 13.83, *SD* = 4.05; *α* = 0.87). Scores were lower for Level 2 (*M* = 9.31, *SD* = 4.82; *α* = 0.91), and for Level 3 (*M* = 10.21, *SD* = 4.77; *α* = 0.90). Similar to the representational artwork, this pattern suggests that viewers engaged with the artworks most strongly at the perceptual level, with engagement decreasing as processing shifted into later levels of processing, and that all three scales demonstrated high reliability within their respective processing stages.

To evaluate whether peoples’ responses to the abstract landscape artwork reflected the structure proposed by the mirror model, we tested the same structural model that was described for the representational artwork. The hierarchical CFA demonstrated acceptable fit overall, with strong performance on incremental fit indices and mixed performance on absolute fit indices. The CFI (0.93) and TLI (0.91) were adequate, suggesting that the hypothesized hierarchical structure captured the majority of systematic covariance among the items. The SRMR (0.05) also indicated a close correspondence between the observed and predicted correlation matrices. The RMSEA value [0.106, 90% CI (0.088, 0.126)] was higher than ideal as well, and we also examined the robust RMSEA for this artwork. The robust RMSEA [0.095, 90% CI (0.070, 0.119)] was slightly lower, suggesting a somewhat improved absolute fit and reducing concerns about overestimating model misfit. The indices suggest a generally well-fitting hierarchical model that provides an adequate representation of participants’ engagement responses to the abstract artwork, even though some residual misfit remains at the item level.

Standardized factor loadings for the lower-order factors were strong and statistically significant throughout the model, supporting the reliability of each level of the mirror model for the abstract artwork. For Level 1, standardized item loadings ranged from 0.73 to 0.82; for Level 2, from 0.80 to 0.89; and for Level 3, from 0.78 to 0.89. These values indicate that the items functioned robustly as indicators of their respective levels. At the higher-order level, all three factors loaded substantially onto the depth of engagement construct (standardized loadings: L1 = 0.87, L2 = 0.82, L3 = 0.76), demonstrating strong shared variance across levels. This pattern supports the idea that while Levels 1–3 are empirically distinguishable, they also operate as interconnected components of a broader, integrative engagement process. Correlations between adjacent lower-order factors reflected similar coupling as with representational art: Level 1 and Level 2 were not significantly negatively related (*r* = −0.09; *p* = 0.18), whereas Level 2 and Level 3 showed a significant positive correlation (*r* = 0.39; *p* = 0.01), consistent with the idea that noticing perceptual details does not imply that a person will further process a work of art, but that when people engage with memory-based processes, they are also more likely to make emotional and evaluative judgments.

The hierarchical structure was also supported for the abstract artwork, again providing evidence for a higher-order depth of engagement construct that organizes responses across the mirror model levels. The abstract artwork yielded a similar pattern of strong higher-order loadings as it was observed for the representational artwork, reinforcing the generality of the depth construct across types of art. Despite this stronger relationship at the hierarchically higher level, the lower-order factors remained distinct, indicating that participants still differentiated among stages of engagement even when the artwork did not provide clear representational perceptual anchors.

### Convergent validity

Correlational analyses provided strong evidence of convergent validity with existing art scales across both artworks. The Aesthetic Experiences Questionnaire showed moderate, positive associations with the AERS depth of engagement global score (representational: *r* = 0.51; abstract: *r* = 0.49) and with each level-specific scale (*r*s = 0.39–0.49), consistent with the expectation that disposition toward aesthetic experiences should predict—but not determine in full—artwork-specific engagement. For the Unusual Aesthetics Experiences Scale scores across both the representational and the abstract artworks, we saw high, positive correlations with the AERS global score (*r* = 0.75 and 0.78, respectively) and with the three level-specific scores (representational: *r*s = 0.59–0.74; abstract: *r*s = 0.59–0.78). Associations were strongest for Level 3 (meaning-making), followed closely by Level 2 and then Level 1, for both artworks, matching theoretical expectations about the overlap between experiences of chills, feeling touched, and absorption assessed by the UAES and higher-level art engagement processes. These results indicate that the AERS aligns well with both trait-level and artwork-specific measures of aesthetic experience, confirming good convergent validity across two distinct artworks.

### Qualitative findings

To supplement our quantitative analyses and shed further light on how aesthetic cognition unfolds through the mirror model, we also examined some of the conceptual features of our text-based AERS responses. In particular, we chose to focus on how the concreteness of ideas and the relatedness of ideas varied across each stage of aesthetic processing.

#### Concreteness

To examine how the semantic qualities of participants’ written responses changed across stages of engagement, we analyzed the concreteness of AERS text responses using established word-level norms. Concreteness scores were drawn from a large-scale database which provides ratings for over 40,000 English word lemmas (Brysbaert et al., 2014). Responses were first lemmatized, each lemma was assigned a concreteness value when available, and concreteness scores were then averaged across all lemmas within a given response. This procedure yielded per-person concreteness scores for each AERS level and artwork, allowing for direct comparison of linguistic abstraction across stages of processing.

Concreteness refers to the extent to which a word denotes a perceptible, tangible object or experience that can be directly observed or otherwise sensed, as opposed to abstract concepts that are intangible, relational, or conceptual in nature (Brysbaert et al., 2014). Higher concreteness values reflect language grounded in sensory and physical experience (e.g., *tree*, *color*, *shape*) and artwork responses such as “The different shades of green in the trees in the foreground caught my eye” (no. 34) whereas lower values indicate more abstract, interpretive, or evaluative language (e.g., *justice*, *love, greatness*) and responses emphasizing subjective meaning, such as “[It] felt like somewhere I used to live, bringing me back to my time in my younger days. A bit of peace and quiet and tranquility” *(no. 140).*

For the representational artwork, a linear mixed-effects model was fit using engagement level as a fixed effect and random intercepts for participants. Fixed effects were evaluated using Type III tests with Satterthwaite degrees of freedom. This revealed a main effect of AERS level on response concreteness, *F*(2, 362.52) = 106.24, *p* < 0.001. *Post hoc* estimated marginal means with Tukey adjustments showed a clear decline in concreteness across levels, with the highest concreteness at Level 1 (*M* = 3.41), followed by Level 2 (*M* = 3.13), and the lowest at Level 3 (*M* = 2.83). All pairwise comparisons were statistically significant (*p*s < 0.001), with standardized mean differences of *d* = 0.72 (Level 1 to Level 2) and *d* = 0.79 (Level 2 to Level 3), indicating that each successive stage of engagement and reflection was associated with a shift toward more abstract language. This pattern is consistent with the mirror model’s proposal that viewers move from perceptual description toward increasingly interpretive and evaluative forms of meaning-making when engaging with representational art.

A similar pattern emerged for the abstract surreal artwork. Again, there was a significant effect of level on concreteness, *F*(2, 374.43) = 61.14, *p* < 0.001. Level 1 responses were the most concrete (*M* = 3.23), followed by Level 2 (*M* = 2.90) and Level 3 (*M* = 2.79). Pairwise comparisons indicated significant decreases from Level 1 to Level 2 and from Level 1 to Level 3 (*p*s < 0.001), as well as a significant difference between Levels 2 and 3 (*p* = 0.028). Compared with the representational artwork, concreteness declined more sharply early in the engagement process (*d* = 0.82) and showed a weaker separation between later stages (*d* = 0.27). This means that participants tended to transition more quickly into abstract language when engaging with the surreal image.

#### Semantic similarity

To examine the conceptual overlap among viewers’ aesthetic responses, semantic similarity was quantified using Latent Semantic Analysis (LSA) mean similarity scores (Landauer et al., 1998). LSA is a corpus-based technique that represents texts as vectors in a reduced semantic space formed from patterns of word co-occurrence. Similarity between two responses is measured via cosine similarity, which ranges from 0 (no meaningful semantic overlap) to 1 (near-identical semantic content), with negative values being rare in practice. Higher similarity scores indicate that responses reference overlapping concepts or themes, even when they use different words. For example, “The calming nature of the grass and sky…” (no. 103) and “Green is soothing… It had a peaceful quality to it,” (no. 35) both describing the painting as peaceful and calming. In contrast, lower similarity scores reflect greater divergence in meaning. For instance, “The different shades of green in the trees…”(no. 34) focus on visual features whereas, “It reminded me of those days back in my country…” (no. 172) refers to an autobiographical memory.

Prior to analysis, all open-ended AERS responses were tokenized and screened to remove excessively short responses that lacked sufficient semantic context; only responses containing at least two words were retained. Singular value decomposition was applied to the resulting corpus to construct the reduced semantic space used for similarity estimation. Cosine similarity was then computed for all possible pairs of responses within each artwork, yielding a dense similarity matrix. Duplicate response pairs, where pairs were reflected (i.e., Response 1, Response 2; Response 2, Response 1) were removed to only keep the first occurrence and ensure observations were independent. To evaluate systematic differences in semantic similarity across engagement levels, separate linear mixed-effects models were estimated for each artwork, with engagement level entered as a fixed effect and random intercepts specified for both responses in each pair. *Post hoc* estimated marginal means and Tukey-adjusted pairwise comparisons were used to assess differences between levels.

For the representational artwork, semantic similarity differed significantly across engagement levels, *F*(2, 52,004) = 3135.80, *p* < 0.001. Estimated marginal means indicated the highest similarity at Level 1 (*M* = 0.43), followed by Level 2 (*M* = 0.33), with the lowest similarity at Level 3 (*M* = 0.29). All pairwise comparisons were statistically significant (all *p*s < 0.001). Level 1 responses demonstrated moderately strong similarity in how participants described the artwork, whereas responses at Level 2 and Level 3 became progressively more different in their semantic content, suggesting that participants tended to talk about increasingly varied ideas as they progressed through the stages of the mirror model.

When the same analysis was run for the surreal abstract artwork, it also revealed a significant effect of engagement level on semantic similarity, *F*(2, 51,369) = 1339.90, *p* < 0.001. Mean similarity was highest at Level 1 (*M* = 0.36), lowest at Level 2 (*M* = 0.27), and intermediate at Level 3 (*M* = 0.30). All pairwise comparisons were statistically significant (all *p*s < 0.001). Surprisingly, unlike the monotonic pattern observed for the representational artwork, Level 3 responses for the surreal image showed greater semantic similarity than Level 2 responses. This inverse-U pattern suggests that while initial perceptual engagement and Level 2 memory-based processing follow the expected trends, there is some factor constraining responses at the third level.

## Study 2

### Methods

#### Participants

300 English-speaking adults were recruited from Prolific.co. Data for two respondents were eliminated for having incomplete responses following checks for low-quality responses (e.g., incomplete responses, duplicates, responses that were returned or denied in Prolific), yielding a final sample of 298 participants that varied in terms of gender (136 women, 159 men, 3 did not disclose/other) and age (*M* = 40.7 years, *SD* = 12.1). Education attainment in the sample ranged from “Less than high school” to “Doctoral or professional degree (e.g., PhD, PsyD, JD, MD)”, but most participants had completed a bachelor’s degree (*Mode* = 109). The sample showed a moderate interest in art (on a 5-point scale, *M* = 2.9, *SD* = 0.75), and 67% of participants had visited an art museum, gallery, or other art exhibit within the last year.

#### Materials

##### Virtual gallery

A 3D, navigable virtual gallery created using the Open Gallery for Arts Research (OGAR) toolkit was deployed for this study (Rodriguez-Boerwinkle et al., 2023) to enable art viewing in a more naturalistic environment than in Study 1. The gallery environment was embedded within the Qualtrics survey and contained three interconnected rooms that displayed 24 artworks in total. To leverage a well-validated environment, the gallery definition (containing the gallery floorplan, artworks, artwork locations, and other associated metadata) used for this study was deployed in two prior virtual gallery studies (Rodriguez-Boerwinkle and Silvia, 2024, 2025).

##### Artworks

The artworks used for this study were obtained from the VAPS (Fekete et al., 2023), described in Study 1. They span a range of sizes, content, styles, and historical periods, but were selected to be half abstract and half representational, given previous research on variable-related differences in aesthetic processing (Bimler et al., 2019; Schepman and Rodway, 2021). Basic artwork information and selection frequencies are available in Supplementary Table 1. For more information about these artworks, the interested reader can see Rodriguez-Boerwinkle and Silvia (2024).

##### Art-Related Scales

Two art-related scales were administered alongside the AERS, the Aesthetic Experience Questionnaire (Wanzer et al., 2020) and an artwork-specific modification of the Unusual Aesthetic Experiences Scale (Silvia and Nusbaum, 2011) following the procedures outlined in Study 1.

##### Personality

The Ten-Item Personality Inventory (TIPI; Gosling et al., 2003), a very brief measure of the Five Factor personality traits, was administered. Reliability measurements in the present sample were acceptable for Openness (*α* = 0.60), Conscientiousness (*α* = 0.68), Extraversion (*α* = 0.73), and Neuroticism (*α* = 0.83), but low for Agreeableness (*α* = 0.45). Because the reliability of the Agreeableness scale was low, the two Agreeableness items were analyzed separately rather than combined into a single score.

#### Procedure

Procedures for Study 2 began with administering our two trait measures, the TIPI and the AEQ, before exposure to the study stimuli. Participants were then directed to visit the navigable OGAR virtual art gallery. After visiting the gallery for as long as they wished, they were instructed to choose an artwork that was meaningful to them. Subsequent scales (the artwork-specific UAES and then the AERS) were administered with reference to the participant-selected artwork. Participants were compensated $7.50 USD for their participation.

## Results and discussion

Descriptive statistics for the full sample are presented in Table 2, and full-sample correlations are shown in Table 4. Since Study 2 participants chose one of 24 possible artworks for the completion of this study, psychometric findings are presented for the full sample, irrespective of whether artworks were abstract or representational in nature.

**Table 4:** Study 2 correlations.

Study Variables	1	2	3	4	5	6	7	8	9	10	11	12	13	14	15
1. Age	-														
2. Gender	−0.11	-													
3. Gallery Visit?	0.16^**^	0.01	-												
4. Art Interest	0.03	−0.07	−0.14^*^	-											
5. Aesthetic experiences (AEQ)	0.11	−0.02	−0.19^**^	0.56^***^	-										
6. Unusual Art Experiences (UAES)	−0.08	0.01	−0.20^***^	0.35^***^	0.54^***^	-									
Big Five traits															
7. O	0.05	0.01	−0.12^*^	0.34^***^	-										
8. C	0.16^**^	−0.01	0.06	0.13^*^	0.28^***^	-									
9. E	0.02	−0.05	−0.08	0.25^***^	0.26^***^	0.25^***^	-								
10. A+	0.04	−0.06	−0.09	0.19^***^	0.16^**^	0.34^***^	0.23^***^	-							
11. A-	−0.19^**^	0.00	0.03	−0.13^*^	−0.28^***^	−0.32^***^	−0.10	−0.30^***^	-						
12. N	−0.05	−0.14^*^	0.04	−0.13^**^	−0.28^***^	−0.47^***^	−0.39^***^	−0.29^***^	0.41^***^	-					
Art Engagement (AERS)															
13. AERS Global	0.10	−0.03	0.04	0.31^***^	0.30^***^	0.25^***^	0.23^***^	0.24^***^	−0.19^**^	−0.24^***^	0.55^***^	0.53^***^	-		
14. Level 1	0.13^*^	−0.03	0.06	0.26^***^	0.26^***^	0.31^***^	0.18^***^	0.17^**^	−0.16^**^	−0.23^***^	0.34^***^	0.32^***^	0.61^***^	-	
15. Level 2	0.04	−0.04	−0.06	0.22^***^	0.23^***^	0.13^*^	0.16^**^	0.15^**^	−0.13^*^	−0.14^*^	0.43^***^	0.37^***^	0.85^***^	0.24^***^	-
16. Level 3	0.10	−0.01	−0.05	0.26^***^	0.22^***^	0.18^**^	0.20^***^	0.24^***^	−0.17^**^	−0.21^**^	0.52^***^	0.55^***^	0.87^***^	0.36^***^	0.64^***^

Notes. O = Openness; C = Conscientiousness; E = Extraversion; A+ = Agreeableness (positively worded item); A- = Agreeableness (negatively worded item); N = Neuroticism. Gender was coded 0 = female; 1 = male. * *p* < 0.05; ^**^ *p* < 0.01; ^***^ *p* < 0.001.

### Artwork choice

Because Study 2 asked people to engage with and answer questions about an artwork from the virtual gallery that was most meaningful to them, we first wanted to examine what choices people made about artworks. Selection frequencies for each artwork are available in Supplementary Table 1. A two-tailed exact binomial test was conducted to determine if participants selected representational artworks more often than abstract artworks, with a hypothesized probability of 0.50 (equal preference). The results indicated that participants chose representational artworks significantly more often than abstract artworks. Of the 298 total choices, 68 (22.8%) were for abstract art, and 230 (77.2%) were for representational art. The difference in proportion was statistically significant, *p* < 0.001. A 95% confidence interval indicated that the true population proportion of choosing abstract art is likely between 18.2 and 28.1%. When asked whether they had seen the artwork they selected before, 206 participants also indicated that they had “definitely not”; only 9 participants reported “definitely yes.”

### Psychometric findings

The composite depth of engagement score averaged 45.61 (*SD* = 8.03) on a 16–60 scale and showed good internal consistency (*α* = 0.85). At the level of the component scales (possible range of 4–20 for each level), Level 1 had a mean of 16.64 (*SD* = 2.68; *α* = 0.75), Level 2 had a mean of 14.22 (*SD* = 3.86; *α* = 0.81), and Level 3 had a mean of 14.74 (*SD* = 3.60; *α* = 0.80). Based on these measures, it seems that people reported moderately high engagement overall, with slightly higher scores for Level 1 compared with Levels 2 and 3, and that each level reliably measured its intended domain of aesthetic reflection.

A hierarchical structural model fit to the AERS data from Study 2 demonstrated adequate or good fit across multiple indices. Fit statistics were: CFI = 0.92, TLI = 0.90, RMSEA = 0.084 [90% CI: 0.069–0.099], and SRMR = 0.05. The CFI and TLI exceeded the conventional threshold of 0.90, and the standardized root mean square residual (SRMR) was below 0.08, indicating reasonable absolute fit. Although the RMSEA was slightly above the conventional 0.08 cutoff, its 90% confidence interval remained within an acceptable range, and overall the model was considered a good representation of the data structure given the theoretical constraints and complexity of the hierarchical specification.

All items loaded significantly onto their intended lower-order factors, and all three lower-order factors loaded significantly onto the higher-order depth of engagement factor. Standardized item loadings ranged from 0.59 to 0.82 for Level 1, 0.68 to 0.79 for Level 2, and 0.55 to 0.82 for Level 3, indicating that each item reliably measured its intended process stage. The lower-order factors themselves showed strong loadings on the higher-order factor (L1 = 0.78, L2 = 0.64, L3 = 0.55), supporting the notion that all three levels collectively contribute to a general depth of engagement construct while retaining level-specific variance. Correlations between the lower-order factors were consistent with our theoretical expectations: Level 1 and Level 2 were negatively correlated (*r* = −0.30), suggesting that participants who reported greater engagement with the early, perceptual aspects of the artwork tended to report lower levels of memory-based processing. Meanwhile, Level 2 and Level 3 were highly positively correlated (*r* = 0.71), indicating that participants who more strongly engaged in memory-based processing were also much more likely to engage in later meaning-making and evaluation.

To examine differences in engagement across the three levels of the AERS, we conducted a linear mixed-effects model, fit with Level as a within-subject factor, artwork type (representational vs. abstract) as a between-subject factor, and random intercepts for participants. Scaled residuals indicated an acceptable fit, and variance components showed meaningful between-participant variability (σ^2^ = 4.93) alongside residual variance (σ^2^ = 6.79). Interestingly, polynomial contrasts revealed significant linear and quadratic components across levels (Level. Linear: *t* = −4.31, *p* < 0.001; Level. Quadratic: *t* = 4.12, *p* < 0.001). This suggests that while there was a general downward trend (linear effect), with lower scores at deeper levels of processing, this decline was not uniform across the levels (quadratic effect). Engagement at Level 2 introduces some variability or disruption that makes aesthetic processing less straightforward, likely prompting responses at Level 3 to partially level off. There was no main effect of artwork type or Level × artwork type interactions (all *p* > 0.70), indicating that the pattern of engagement across stages was similar for abstract and representational works.

Post-hoc estimated marginal means contrasts confirmed these patterns. For both abstract and representational artworks, Level 1 engagement was significantly higher than Level 2 (*p* < 0.001) and L3 (*p* < 0.001). Differences between Level 2 and Level 3 were not statistically significant for either artwork type (abstract: *p* = 0.33; representational: *p* = 0.11), which means that while initial perceptual engagement tends to be higher, the later stages of intermediate, memory-based processing and late-stage meaning-making are roughly equivalent in terms of their average score.

### Convergent and discriminant validity

To evaluate convergent and discriminant validity, we examined whether AERS scores were more strongly associated with theoretically related measures of aesthetic engagement than with broader personality traits. Stronger relationships with the art-related measures, alongside comparatively modest relationships with the Big Five traits, supported this expected pattern. Correlational analyses examined how personality traits and aesthetic predispositions were associated with AERS engagement scores (see Table 4). Across the Big Five, openness showed the most consistent positive associations with AERS scores, correlating moderately with the global depth of engagement score (*r* = 0.30) and with each level of engagement (L1: *r* = 0.26, L2: *r* = 0.23, L3: *r* = 0.22), aligning with well-established evidence that individuals high in openness tend to seek, tolerate, and engage deeply with complex aesthetic experiences (Afhami and Mohammadi-Zarghan, 2018; Fayn et al., 2015; Silvia et al., 2015). Conscientiousness (*r* = 0.25) and extraversion (*r* = 0.23) showed smaller, but still positive associations with global engagement, with conscientiousness being linked most strongly to early, automatic perceptual engagement (*r* = 0.31). Agreeableness, measured via two single items, showed positive associations for the positively worded item (“Sympathetic, warm”; *r* = 0.23) and weaker negative associations (“Critical, quarrelsome”; *r* = −0.19) for the reverse-scored item.

In contrast, art-related measures showed substantially stronger relationships with AERS scores. The AEQ composite was strongly correlated with the global AERS score (*r* = 0.55), with moderate-to-strong associations across all levels (L1: *r* = 0.34, L2: *r* = 0.43, L3: *r* = 0.52). Similarly, the artwork-specific UAES demonstrated robust associations with global engagement (*r* = 0.53) and each level score (L1: *r* = 0.32, L2: *r* = 0.37, L3: *r* = 0.55). These strong correlations indicate that individuals who are dispositionally inclined toward aesthetic experiences, or who report atypical aesthetic experiences in relation to specific works also tend to show deeper engagement with artworks along the dimensions measured by the AERS, broadly supporting the convergent validity of the scale.

### Qualitative findings

As in Study 1, linguistic concreteness was examined to assess whether participants’ open-ended written responses reflected systematic shifts in the type of language used across levels of aesthetic engagement. Semantic similarity was not examined for Study 2, because the study design allowed participants to select their own meaningful artwork from a larger pool, resulting in responses that naturally varied widely in content.

Using the same procedure as in Study 1, concreteness scores were assigned at the lemma level and averaged within each participant’s response for each AERS level, yielding per-level concreteness scores nested within individuals. A linear mixed-effects model with Level as a fixed effect and random intercepts for participants revealed a significant main effect of Level on mean concreteness, *F*(2, 588.61) = 86.54, *p* < 0.001. Estimated marginal means indicated a steady decrease in concreteness across levels, with the highest concreteness at Level 1 (*M* = 3.22), followed by Level 2 (*M* = 3.05), and the lowest concreteness at Level 3 (*M* = 2.89). *Post hoc* pairwise comparisons using Tukey adjustment confirmed that all levels differed significantly from one another (all *p*s < 0.001). Responses at Level 1 were significantly more concrete than those at Level 2 and Level 3, and Level 2 responses were also more concrete than Level 3 responses, replicating the pattern observed in Study 1 across both artworks in a broader, participant-chosen set of meaningful art images.

## General discussion

We have presented two studies that developed and provided initial testing of the AERS, a new measure assessing the intensity and quality of engagement in the process of art appreciation. The two studies provide converging evidence that the AERS captures cognitive aspects of aesthetic engagement that are supported by and interpretable under the mirror model of art (Tinio, 2013). Study 1 tested the scale across two stylistically-distinct landscape artworks (a representational image and a more abstract, surreal image) and Study 2 was conducted in a more naturalistic art-viewing context in which participants freely explored a virtual gallery and selected an artwork that was personally meaningful to them. Both studies found strong evidence for the hierarchical structure of the scale. Three distinguishable lower-order levels correspond to early automatic processing, intermediate, memory-based processing, and meaning-making and final judgment, which inform a higher-order depth of engagement construct. In both studies, the pattern of strong item loadings, robust higher-order factor loadings, and theoretically-consistent correlations between adjacent levels suggest that the AERS successfully operationalizes art engagement as a staged process where people tend to progress through distinct levels of processing but may revisit one or another.

The two studies also offer evidence for the interpretative validity of the AERS. Quantitative results showed graded engagement across levels, with the strongest endorsement at the perceptual stage and more attenuation at later stages, which is in line with the idea that low-level perceptually focused processing is less demanding than high-level conceptual and thematic judgments (Tinio, 2013). Importantly, this pattern held across both artworks selected by the researcher in Study 1 and artworks that participants considered personally meaningful in Study 2. Engagement does not decline uniformly across levels. Instead, intermediate, memory-based processing (Level 2) and final meaning-making (Level 3) level off, consistent with the idea that final aesthetic judgments can build upon, but are not necessarily the outcome of, early perceptual exploration. The absence of any main effects or interactions involving artwork type suggests that this pattern reflects a general property of aesthetic engagement rather than features specific to abstract or representational art.

Adding to the results of rating scales measuring the extent of art engagement, qualitative analyses provide information about the nature of the engagement at three levels of processing. Both studies showed that linguistic concreteness in participant reflections decreased across engagement levels (regardless of the kinds of artworks), meaning that peoples’ open-ended responses moved from perceptually-focused descriptions of objects or features they could see on the canvas toward increasingly abstract and evaluative language as hypothesized in the mirror model of art (Tinio, 2013). For example, when one participant (no. 24) in Study 1 provided open-ended responses for Roberto Matta’s *Invasion of the Night*, they started by mentioning the visual features they saw: “Mainly yellow and blue hues create a bright impression on first glance. However, the eye is then drawn to the more reddish elements and this creates a dissonance of sorts.” The participant went on to describe what the work reminded them of using a mix of more concrete and abstract ideas, stating, “It reminded me of works that I have seen by surrealist artists, such as Dali whose museums in Spain I have visited. There is imagery reminiscent of natural bodily features,” before concluding, in very abstract terms, that the painting “evokes feelings of death and despair.”

Semantic similarity analyses in Study 1 revealed similar patterns to concreteness scores: early-stage responses were more similar across viewers, whereas later stages became more divergent, perhaps reflecting person-specific meaning-making. To illustrate this, we offer open-ended responses from another participant from Study 1(no. 64) reacting to *Invasion of the Night.* Similar to the response of participant (no. 24) in the previous paragraph, they first noted sensory elements of the artwork: “The bright yellows and reds drew my attention first. The colors stood out to me, and then I saw the shapes, and it reminded me of something I had seen before.” Then, the two participants diverge, with the first relating the artwork to other surrealist pieces they saw in their travels while the second takes a different (and more lighthearted) turn, musing: “I thought of an episode of SpongeBob where we get a look into SpongeBob’s brain. The inside of his mind reminds me of the different sections of the artwork”, before deciding that the artwork ultimately reflected themes “about how the mind works. And the many ways that [art] can be interpreted.”

The two studies support convergent validity of the AERS. Scores are related to both dispositional and artwork-specific measures of aesthetic experience. The AERS shows moderate associations with the dispositional measure of aesthetic experience, which describes people’s tendency to have intense emotional and cognitive experiences, as well as appreciate art in a flow-like manner (Wanzer et al., 2020). Flow is described as the optimal experience (Csikszentmihalyi, 1997), stressing similarity with the deep engagement. Those who are more deeply engaged with a specific work of art as measured by the AERS in general tend to focus on the subtle aspects of art, they try to understand what the artist is trying to communicate, ultimately feel moved, and relate art to their knowledge of historical time or current culture.

This result fits into the larger literature on personality traits and prediction of everyday behavior, which finds that traits reliably predict behavior that is conceptually aligned with those traits. For example, rich literature exists documenting that Big Five personality traits predict a broad range of behaviors, from the number of parties people attend, how regularly they physically exercise, how often they play musical instruments, to the length of their text messages (e.g., Ozer and Benet-Martínez, 2006; Paunonen, 2003; Stachl et al., 2020). There is also extensive research focusing on trait prediction of specific classes of behavior, such as creative activities (Puryear et al., 2017), physical activity (Sutin et al., 2016), or academic achievement (Noftle and Robins, 2007). Along the same lines, it makes sense that the dispositional tendencies toward typical (trait-like) aesthetic engagement that resemble optimal experience of flow (measured by the AEQ) predict how people actually engage with artworks in specific moments. Whereas most prior research has focused on the core personality traits (e.g., Big Five trait domains and their aspects), the present research extends the evidence for trait prediction of conceptually relevant behavior to surface traits, such as aesthetic experience (as discussed by Asendorpf and Van Aken, 2003; Lebuda, 2025; Lebuda et al., 2021).

Further supporting convergent validity, both studies showed associations between the AERS and unusual aesthetic experiences (Silvia and Nusbaum, 2011). This finding indicates that those who engage with an artwork more deeply across levels of aesthetic processing are more likely to have experiences like getting goose bumps, feeling touched, being absorbed or immersed in the experience, and feeling awe and wonder. Importantly, the association between unusual aesthetic experiences and art engagement was higher for processing beyond the perceptual level, suggesting that active cognitive processing and meaning-making, rather than what is seen in concrete, sensorial terms, is what largely contributes to experiences of absorption or enraptured emotions during art engagement. Furthermore, this connection between deep engagement with art and unusual aesthetic experiences is evident in people’s reflections. For example, one participant who reported a particularly deep experience with the artwork wrote, “The art style and what it was depicting was so real and heart wrenching…[it] gave me chills” (no. 173).

## Limitations and future directions

In our effort to study the complex, unfolding cognitive process of art engagement in a controlled but ecologically informed way, using a new measure created for this purpose, several limitations and directions for future research are worth highlighting.

First, although the two studies sought to offer a progressively more ecologically valid setting, both studies in the present paper were conducted in online settings. Study 1 relied on researcher-selected artworks, which allowed control of stimulus content (representational and abstract) and facilitated direct comparisons across participants. Study 2 moved toward greater ecological validity by embedding the task in a navigable online gallery and allowing participants to select artworks that were personally meaningful, thus creating circumstances a step closer to those stimulating engagement in everyday gallery or museum settings. Although the latter design better approximates naturalistic art engagement, the gallery was not curated in the traditional sense of presenting a unified theme. This was a deliberate choice in order to avoid some curatorial effects, but it represents a notable departure from everyday art settings. Future work should extend the ecological validity of this research by bringing it into physical museum and gallery contexts, where spatial layout or curatorial intent may shape engagement (Illes and Tinio, 2022; Szubielska et al., 2021). Prior research suggests that many aspects of aesthetic experience are comparable across digital and physical settings, but meaningful differences can emerge depending on context (e.g., Darda et al., 2025; Rodriguez-Boerwinkle et al., 2023; Specker et al., 2017). A key advantage of the AERS that can contribute to better understanding of the context differences in art experience is its ability to capture not only the degree of engagement, but also its nature, through open-ended responses about engagement at different levels of processing.

A second limitation concerns the reliance on self-report to measure internal engagement processes. The validity of the responses depends on participants’ ability to introspect and reflect on their experience. Although Level 1 engagement is theoretically defined as rapid and partly implicit, participants consistently report high Level 1 engagement across artworks and studies. However, these reports might not fully correspond to these early processes, as they occur outside of awareness. Instead, AERS scores, and especially the open-ended reflection, likely reflect recalled (and thus partly reconstructed) engagement after-the-fact rather than the full stream of cognitive processing as it unfolds. This limitation points directly to future need for multi-method validation. Combining the AERS with eye-tracking measures or neuroscientific methods (e.g., EEG or fMRI) would allow researchers to examine how reflective reports align with real-time indicators of processing. In this way, the AERS can function as a bridge between subjective experience and other markers of processing. An alternate line of research might include systematically examining the depth and quality of engagement offered by participants in response to being allowed to view works of art for experimentally manipulated timeframes. This may offer insight into the concrete, temporal aspects of art engagement.

Relatedly, by operationalizing the mirror model’s stages as separable but ordered dimensions, the AERS necessarily imposes structure on what is likely a fluid and recursive process. Although the present findings support graded engagement and partial coupling between stages, a scale cannot fully capture idiosyncratic trajectories, moment-to-moment transitions, or rare and unusual experiential patterns. For example, a viewer might initially interpret an ambiguous figure in an artwork as threatening, connect it to a personal memory, and form an overall judgment about the work, only to notice a previously overlooked visual detail that changes the initial perception and prompts a new interpretation. In this case, engagement would move recursively between perceptual, memory-based, and meaning-making processes rather than unfolding in a strictly linear sequence.

One final limitation is methodological in nature. The AERS was administered after a scale assessing typical aesthetic experiences when engaging with art. This was a deliberate choice because aesthetic experiences are measured as trait-like engagement and as such represent a personal attribute with which people enter a specific encounter with a piece of art. However, it is possible that completing this measure before the AERS primed participants to consider attributes of perceptual, emotional, and cognitive engagement that otherwise they could not have considered before they got to the AERS and thus showed higher scores than they would otherwise. Future research should investigate such possible priming effects by administering the AERS immediately after or along with art experience.

The present work should be understood as an early step in a cumulative program of construct validation. As emphasized in broader discussions of scale development, construct validation is an incremental process that unfolds across studies, samples, and methods (Smith, 2005). Several fruitful directions follow from this logic. Future studies could compare engagement across online and in-person environments, examine differences between art novices and experts, or test how familiarity with artistic styles or cultural traditions shapes engagement, all of which have been previously related to art experience outcomes (Augustin and Leder, 2006; Darda et al., 2025; Song et al., 2021). Future research should also examine more in-depth the qualitative responses on the AERS. Here we primarily focused on the psychometric properties and initial testing of mirror model propositions, but analyses can also focus on open-ended questions across levels of processing. For instance, existing research shows that art can evoke many emotions (Silvia, 2009) and that people differ in emotional diversity in encounters with art (Rodriguez-Boerwinkle and Silvia, 2024). AERS can provide information about the nature of emotion processing and enable us to answer questions such as whether people start with more general or basic feelings and proceed to more nuanced and granular ones.

Finally, because the AERS is designed to capture engagement as a process rather than a disposition or outcome, it is particularly well suited for testing hypotheses about the mechanisms of cognitive and emotional effects of art. A growing body of work proposes that art engagement can foster outcomes such as creative thinking (Tang et al., 2025), well-being (Cotter et al., 2024; Trupp et al., 2025), or empathy (Kou et al., 2020), but often without specifying how these effects arise. A process measure allows researchers to test whether such benefits depend on the depth or configuration of engagement across stages. For instance, cognitive benefits may emerge only when viewers move beyond perceptual analysis into deeper sustained processing (e.g., Ishiguro and Okada, 2021). In this sense, the AERS not only fills a measurement gap, but also allows for more precise theorizing about when and how aesthetic experiences result in broader psychological effects.

## Impacts and conclusion

In addition to its theoretical contributions, the AERS provides a practical tool for museums, galleries, educators, and arts organizations seeking to understand how visitors engage with works of art. Existing visitor evaluation tools often focus on satisfaction, enjoyment, or time spent in exhibits. The AERS instead provides theoretically-grounded information about perceptual, associative, and meaning-making dimensions of visitor engagement. Using the AERS would allow institutions to evaluate whether exhibitions, interpretive materials, educational programs, or digital interventions support particular forms of engagement rather than simply determining whether visitors liked an experience. For example, a museum evaluating competing options for a set of new artwork labels might administer the AERS and find that visitors who receive one set of labels more frequently connect the work to personal memories or broader social issues. Such a finding would provide concrete guidance about which labels are better for supporting the museum’s educational goals. In this way, information collected using the AERS could inform exhibition design, museum education, audience development, and evaluation of arts-based interventions in health and wellbeing contexts.

## Data Availability

The raw data supporting the conclusions of this article will be made available by the authors, without undue reservation.

## Footnotes

1 Other widely used instruments, such as the *Aesthetic Fluency Scale* (Cotter et al., 2023; Smith and Smith, 2006) and *VAIAK* (Specker et al., 2020), are not included here because they assess art knowledge or interpretive skill rather than aesthetic experience per se.
