# Supplementary material for: Development and validation of the Art Engagement Reflection Scale
Source: Front Psychol. 2026 Sep 22;17:1794908. doi: 10.3389/fpsyg.2026.1794908 (PMC13638572; doi:10.3389/fpsyg.2026.1794908)
Supplement: Supplementary file 1 [file Supplementary_file_1.docx]

**Supplementary Materials**

**Appendix 1**

**The Art Engagement Reflection Scale**

*This scale is intended to measure the process of cognitive engagement carried out during art viewing.*

*Factors reflect three levels of engagement, Early, Automatic Processing, Intermediate, Memory-Based Processing, and Meaning Making and Final Judgement, described by the Mirror Model of Art (Tinio, 2013). These factors can be combined to form a higher-order factor, Depth of Engagement.*

*For administration, the administrator is encouraged to use the scale modularly to fit their needs. For example, a researcher can elect to use qualitative and quantitative items from only one level of the scale, or they may wish to use the quantitative or qualitative item sets alone, foregoing a mixed-methods approach.*

**Level 1: Early, Automatic Processing**

**Quantitative (Likert items):**

*Please rate the extent to which you agree with the following statements based on your initial reaction to the artwork. (1 = Strongly Disagree, 5 = Strongly Agree)*

"I was immediately drawn to specific visual details in the artwork (e.g., colors, shapes, textures)."

"Certain elements of the artwork stood out to me as soon as I looked at it."

"I noticed the fine details of the artwork quickly."

"The textures or patterns in the artwork caught my eye right away."

**Qualitative (Open-ended question):**

"Describe the specific visual elements (e.g., colors, textures, shapes) that first caught your attention. What was it about them that stood out to you?"

**Level 2: Intermediate, Memory-Based Processing**

**Quantitative (Likert items):**

*Please rate the extent to which you agree with the following statements based on your reflections after observing the artwork for a few moments. (1 = Strongly Disagree, 5 = Strongly Agree)*

"The artwork reminded me of a past experience or memory."

"As I looked at the artwork, I found myself making connections with other things I’ve seen or learned."

"Over time, my understanding of the artwork deepened."

"I found myself reflecting on how the artwork relates to my personal experiences."

**Qualitative (Open-ended question):**

"What specific memories, prior knowledge, or personal experiences came to mind as you viewed this artwork? How did they influence your understanding of the art?"

**Level 3: Late, Meaning-Making Processing**

**Quantitative (Likert items):**

*Please rate the extent to which you agree with the following statements based on your final thoughts and feelings about the artwork. (1 = Strongly Disagree, 5 = Strongly Agree)*

"The artwork conveyed meaningful themes or ideas to me."

"I experienced strong emotions while viewing the artwork."

"My overall impression of the artwork changed as I thought more about it."

"The artwork made me reflect on deeper concepts or questions."

**Qualitative (Open-ended question):**

"What broader themes, ideas, or emotions did the artwork evoke for you? How did your interpretation of the artwork evolve as you spent more time with it?

**Supplementary Table 1.**

*Basic Artwork Information for Study 1 and Study 2*

| VAPS # | Art Name | Artist | Year | Representational? | Selection Frequency |
| --- | --- | --- | --- | --- | --- |
| Study 1 (n = 192) | | | | | |
| 30411 | Houses near Orleans | Camille Carot | 1830 | Yes | 192 |
| 31105 | Invasion of the Night | Roberto Matta | 1941 | No | 192 |
| Study 2 (n = 298) | | | | | |
| 20128 | Sommer | Arcimboldo, Giuseppe | 1563 | Yes | 14 |
| 20309 | Portrait of a Black Man | Gericault, Theodore | 1822-23 | Yes | 13 |
| 50833 | Abstract Composition | Wadsworth, Edward | 1915 | No | 3 |
| 50703 | Rising Sun | Klee, Paul | 1919 | No | 7 |
| 51002 | Lake George, Coat and Red | O’Keefe, Georgia | 1919 | No | 3 |
| 10515 | The Pirates | Slevogt, Max | 1914 | Yes | 15 |
| 51104 | Man Looking at Woman | Gottlieb, Adolph | 1949 | No | 1 |
| 50811 | The Passage from Virgin to Bride | Duchamp, Marcel | 1912 | No | 4 |
| 30221 | Vienna seen from the Belvedere | Canaletto/Bellotto, Bernardo | 1758 | Yes | 33 |
| 20605 | Woman with a Parasol | Signac, Paul | 1893 | Yes | 6 |
| 41024 | Skull with Candle | Richter, Gerhard | 1983 | Yes | 25 |
| 50901 | Composition | Lissitzky, El (Eliezer) | 1922 | No | 4 |
| 31017 | Subway | Estes, Richard | 1969 | Yes | 17 |
| 11026 | Barbecue | Fischl, Eric | 1982 | Yes | 25 |
| 51326 | Lysander I | Olitski, Jules | 1970 | No | 4 |
| 51115 | Bather | Miro, Joan | 1932 | No | 1 |
| 11019 | Repression | Rivera, Diego | 1931 | Yes | 39 |
| 51301 | Quattro Stagioni, Autunno | Twombly, Cy | 1993-95 | No | 11 |
| 51215 | Soul of the Underground | Dubuffet, Jean | 1959 | No | 4 |
| 50940 | Praise I | Riley, Bridget | Unknown | No | 4 |
| 31103 | Blood with Tell | Magritte, René | 1959 | Yes | 28 |
| 40704 | Still Life with Flowers and Oranges | Jawlensky von, Alexej | 1909 | Yes | 5 |
| 40405 | Dessertfrüchte mit  Elfenbeinhumpen | Preyer, Johann Wilhelm | 1838 | Yes | 10 |
| 51013 | Dr Feel Good – Clermont Ferrand | Villeglé de la, Jacques | 2000 | No | 22 |

*Notes.* All participants in Study 1 saw both available artworks. Participants in Study 2 were asked to select one of the available 24 artworks. Artwork images were obtained from the Vienna Art Picture System and can be obtained at the request of the database authors using the provided VAPS # codes.
